# Therapeutic effects of Tetanus neurotoxin in spinal cord injury: a case series on four dogs

**DOI:** 10.1038/s41394-020-0258-9

**Published:** 2020-02-17

**Authors:** Stefan Hesse, Anna Kutschenko, Beatrice Bryl, Martin Deutschland, David Liebetanz

**Affiliations:** 1 Neurological Department, Medical Park Berlin Humboldtmühle, Berlin, Germany; 2 Department of Clinical Neurophysiology, University Medical Centre, Göttingen, Germany; 3 Neurological Referral Veterinary Surgery, Berlin, Germany; 4 Present Address: Department of Neurology, Hannover Medical School, Hannover, Germany

**Keywords:** Spinal cord diseases, Trauma

## Abstract

**Study design:**

Case series on four dogs.

**Objectives:**

To determine the alleviation of motor symptoms in spinal cord injury (SCI) by tetanus neurotoxin (TeNT).

**Setting:**

Different Berlin veterinary clinics, Germany.

**Methods:**

We report on the effect of intramuscular injections of low-dose TeNT into paretic hind limb muscles 2–157 weeks after SCI due to lumbar disc herniation in a clinical case series on four dogs. All dogs underwent unsuccessful or incomplete surgical decompression prior to TeNT treatment. TeNT was injected on a compassionate basis. Stance, gait ability and the diameter of the rectus femoris muscle were assessed as parameters.

**Results:**

All four dogs improved their stance and three of these dogs improved in gait at 4 and 6 weeks after TeNT injections without evidence of side effects or spreading of TeNT effects. At the same time, the size of the rectus femoris muscle diameter increased considerably as compared with baseline (baseline: 100%; 4 weeks: 148.7% ± 10.9%; 6 weeks: 137.1% ± 7.9%).

**Conclusions:**

Facilitation of α-motor neurons by TeNT injections into paretic hind limb muscles of four dogs improved standing and/or gait abilities and partly reversed muscle atrophy after SCI. The absence of generalized or painful muscle spasms supports the safety of low-dose TeNT. Therefore, TeNT might evolve as a promising therapeutic option for muscle paresis of central origin, e.g. in individuals with SCI, stroke or multiple sclerosis.

## Introduction

A major therapeutic goal after spinal cord injury (SCI) is the restoration of standing and walking to improve the ability to perform activities of daily living and to improve quality of life [1–3]. To this end, experimental approaches aim at restoring the activity of depressed spinal locomotor central pattern generators (CPG) by electrical stimulation, by intraspinal pharmacological treatment or by a combination of both [2, 4]. However, the translation into the clinical setting still faces enormous technological challenges [5–7].

Despite intensive research during recent decades, there is still a need for novel treatments to restore motor function after SCI. In line with this strategy, the present case series introduces tetanus neurotoxin (TeNT) as a potential pharmacological treatment for the restoration of spinal motor circuits. TeNT is a 150 kDa protein that is produced by the anaerobic bacterium *Clostridium tetani*. TeNT binds to the presynaptic membrane of motor neurons from where it is transported retroaxonally to the spinal cord. After transcytosis into spinal inhibitory interneurons, TeNT cleaves VAMP (vesicle-associated membrane protein)/synaptobrevin, a protein of the SNARE (soluble N-ethylmaleimide-sensitive factor attachment protein receptor) complex of the neurotransmitter containing synaptic vesicle. Thereby, it blocks the release of the inhibitory neurotransmitters glycine and GABA (gamma-aminobutyric acid). This leads to a disinhibition and consequently to a pronounced facilitation of motor neuron activity [8, 9].

Naturally emerging generalized tetanus results from wound infections with *Clostridium tetani* and subsequent TeNT intoxication, leading to uncontrolled generalized muscle spasms, rigidity, and autonomic symptoms usually beginning in the bulbar muscles [10, 11]. However, injections of very low doses of TeNT are able to induce a dose-dependent, localized, and temporary increase of muscle tone [12]. Because of its unique mode of action on the facilitation of spinal motor neurons, low-dose TeNT injections have been proposed as a possible therapeutic option for centrally originated muscle weakness [12–16].

Due to biomechanical constraints, chondrodystrophic canine breeds are highly susceptible to disc herniations [17, 18]. Since the canine spinal cord extends up to the 6th lumbar vertebra, severe lumbar disc herniations regularly result in a paraplegia rather than a caudal syndrome, which would be expected in humans [17, 19].

Here, we report for the first time on therapeutic effects of low-dose TeNT injections into paretic muscles in dogs with SCI. Four dogs suffering from paraplegia due to disc herniation of the thoracolumbar spine were treated with intramuscular TeNT injections on a compassionate base. All dogs had a history of immediate and partly even long-term unsuccessful or incomplete surgical intervention after SCI, which means no improvement of clinical outcome following the surgery. Effects of TeNT injections on gait and stance as well as on the thickness of the injected muscles were assessed 4 and 6 weeks following the injections.

## Materials and methods

### Selection of animals

Four dogs (referred to as dog #1–dog #4) with SCI were referred from different Berlin veterinary clinics. The patient owners signed an informed consent for each dog regarding the compassionate use of low-dose intramuscular injections of TeNT. The treatment of the dogs was conducted in accordance with German animal protection laws and was verbally approved by the Institute of Animal Welfare of the Department of Veterinary Medicine at Free University of Berlin. Ranging from 2 to 157 weeks before the TeNT injection, the selected animals had a history of non-traumatic disc herniation at level T13/L1 or L1/L2 resulting in SCI, which was confirmed by spinal NMR (nuclear magnetic resonance). Clinically, the animals suffered from persistent paraplegia, para- or monoparesis. Prior to study participation they had undergone operative decompression within 1–52 weeks after SCI and participated in subsequent physiotherapy (Table 1). Other causes of the dogs’ hind limb paresis, such as additional radiculopathies, were excluded by bilateral electromyographic needle investigations of the gluteus medius, quadriceps femoris, gastrocnemius and tibialis cranialis muscles.

**Table 1:** Animal data, disease history and TeNT injection scheme.

Dog no.	Age (years)	Weight (kg)	Diagnosis	Interval SCI to surgery (weeks)	Interval SCI to TeNT (weeks)	Injected muscles	Dosage TeNT (pg)
#1	11	18	Paraplegia sub L1 No nociception No proprioception No urine and stool control	52	83	M. gluteus medius (bilateral) M. vastus medialis (bilateral) M. vastus lateralis (bilateral) M. gastrocnemius (lateral and medial head, bilateral)	375 pg per muscle = 3750 pg total
#2	6	7	Monoparesis right side (drop paw) Preserved nociception Incomplete proprioception Unrine and stool control	1	2	M. tibialis cranialis (right) M. quadriceps (right)	187.5 pg M. tibialis cranialis 125 pg M. quadriceps = 312.5 pg total
#3	3	8	Left-accentuated paraparesis sub L1 No nociception Incomplete proprioception No urine and stool control	3	157	M. gluteus medius (bilateral) M. vastus medialis (bilateral) M. vastus lateralis (bilateral) M. gastrocnemius (lateral and medial head, bilateral)	187.5 pg (right) and 250 pg (left) per muscle = 2187.5 pg total
#4a	15	11	Right-accentuated paraparesis sub Th13 No nociception Incomplete proprioception No urine and stool control	1	7	M. gluteus medius (bilateral) M. vastus medialis (bilateral) M. vastus lateralis (bilateral) M. gastrocnemius (lateral and medial head, bilateral)	250 pg (right) and 187.5 pg (left) per muscle = 2187.5 pg total
#4b	15	11	Right-accentuated paraparesis sub Th13 No nociception Incomplete proprioception Urine and stool control	1	23	M. gluteus medius (bilateral) M. vastus medialis (bilateral) M. vastus lateralis (bilateral) M. gastrocnemius (lateral and medial head, bilateral)	250 pg (right) and 187.5 pg (left) per musle = 2187.5 pg total

Four different dogs (dog #1–dog #4) were injected with individualized TeNT injection protocols. Dog #4 was injected twice (dog #4a and dog #4b) with an interval of 16 weeks in between.

Proprioception and nociception of each dog were evaluated before TeNT injections. This was performed through examination of postural reactions and sensation of the claws. Proprioceptive positioning is a test designed to evaluate the conscious awareness of the limb position and its movement. To this end, the dog’s foot was passively turned over so that its dorsal surface had contact with the ground. With normal proprioception, the dog would immediately reposition its foot. Sensation of pain was tested by pressuring the dogs’ nail bed of the claw and observing the consecutive reaction.

### Intramuscular injection of low-dose TeNT

Toxin aliquots (5 ng TeNT/ml) for the present case series were provided by courtesy of Dr. Andreas Rummel (Institute of Toxicology, Hannover Medical School, Hannover, Germany). Immediately before the injection, one vial (stored at −20 °C) was thawed at room temperature and diluted in PBS (phosphate buffered saline) with 0.1% BSA (bovine serum albumin) to a final concentration of 625 pg TeNT/ml.

The injected TeNT dose was calculated from a preceding in vivo mice study. This study quantified TeNT induced focal increase in muscle tone using an automated running wheel paradigm [12]. First of all, the injected dose of TeNT for the dogs was calculated based on their weight in the ratio to the mice’s weight. Depending on the motor deficits of the dogs (Table 1) the injected TeNT dose was adapted and muscles were chosen. Using a 30 G needle, volumes between 200 and 600 µl per muscle were injected. Dog #1, dog #3 and dog #4 received bilateral injections into the gluteus medius, vastus medialis, vastus lateralis, and the medial and lateral head of the gastrocnemius muscle. Dog #4 was treated twice with a time interval of 16 weeks between the first and second injection referred to as dog #4a and dog #4b. Dog #2, which predominantly suffered from a drop paw of the right hind limb, received TeNT injections only into the right tibialis cranialis muscle and the quadriceps femoris muscle.

For the ultrasound-guided intramuscular injection procedure, animals were sedated with intravenous Dexmedetomidin-hydrochlorid (Dexdomitor, Orion Pharma, Finland) in a dose of 5 µg per kg body weight, combined with Butorphanol (Alvegesic, Virbac, Switzerland) in a dose of 100 µg per kg body weight.

### Assessment of side effects of TeNT

After TeNT injections, the dogs were visited at weeks 4 and 6. At these visits, the dogs were assessed for painful muscle spasms at rest as well as during movement. Beyond that, the dog’s owners were queried whether they had noticed painful muscle spasms during the previous interval before the current visit. Furthermore, all dogs were clinically examined at both visits to be able to detect TeNT effects in other regions than the injected muscles.

### Assessment of effects of TeNT on stance and gait

The primary variable was the standing and walking ability assessed with the help of the functional scoring system in dogs (FSSD) [19] that was slightly modified since each hind limb was rated separately (modified functional scoring system in dogs = mFSSD). Every dog was videotaped for 30 s from both sides and from behind when standing or walking on a non-slippery surface. Dogs that could not bear weight on their pelvic limbs were also videotaped either when crawling or being supported by a sling to allow non-weight-bearing voluntary movements of their pelvic limbs. The dogs were scored by a physiotherapist, who was not involved in the experiments and unaware of the protocol applied.

The mFSSD distinguishes five stages of recovery (Table 2): paralysis with no voluntary pelvic movements (stage 1), non-weight-bearing voluntary pelvic movements (stage 2), voluntary pelvic movements with occasional weight-bearing steps (stage 3), weight-bearing movements all the time with decreased motor strength (stage 4), and normal motor strength with pelvic limb ataxia (stage 5). Each of the stages was subdivided into three inter stages, resulting in a scale from 0 (no pelvic limb movement and no deep pain sensation) to 14 (normal pelvic limb gait). This means the higher the scale the better the recovery [19].

**Table 2:** Modified functional scoring system in dogs (mFSSD).

Stages of recovery	FSSD score	Description of pelvic limb gait
Stage 1 (paralysis with no voluntary pelvic movements)	0	No pelvic limb movement and no deep pain sensation
	1	No pelvic limb movement with deep pain sensation
	2	No pelvic limb movement but voluntary tail movement
Stage 2 (non-weight-bearing voluntary pelvic movements)	3	minimal non-weight-bearing protraction of the pelvic limb (movement of 1 joint)
	4	Non-weight-bearing protraction of the pelvic limb with > 1 joint involved < 50% of the time
	5	Non-weight-bearing protraction of the pelvic limb with > 1 joint with > 50% of the time
Stage 3 (voluntary pelvic movements with occasional weight-bearing steps)	6	Weight-bearing protraction of the pelvic limb < 10% of the time
	7	Weight-bearing protraction of the pelvic limb 10–50% of the time
	8	Weight-bearing protraction of the pelvic limb > 50% of the time
Stage 4 (weight-bearing movements all the time with decreased motor strength)	9	Weight-bearing protraction 100% of the time with reduced strength of pelvic limb. Mistakes > 90% of the time (e.g., crossing of pelvic limbs, scuffing foot on protraction, standing on dorsum of foot, falling)
	10	Weight-bearing protraction of pelvic limb 100% of the time with reduced strength. Mistakes 50–90% of the time
	11	Weight-bearing protraction of pelvic limb 100% of the time with reduced strength. Mistakes < 50% of the time
Stage 5 (normal motor strength with pelvic limb ataxia)	12	Ataxic pelvic limb gait with normal strength, but mistakes > 50% of the time (e.g., lack of coordinatino with thoracic limb, crossing of pelvic limbs, skipping steps, bunny-hopping, scuffing foot on protraction)
	13	Ataxic pelvic limb gait with normal strength, but mistakes < 50% of the time
	14	Normal pelvic limb gait

Each hind limb is rated separately using the mFSSD. The five stages of recovery with their particular three subdivisions are presented resulting in a score from 0 to 14 to assess pelvic limb gait and weight bearing after SCI in dogs [19].

### Assessment of effects of TeNT on muscles thickness

The bilateral thickness (cm) of the rectus femoris muscle was measured by ultrasound (Esaote, MyLab; 6 MHz) immediately before the injection, at weeks 4 and 6. The ultrasonic probe was positioned on the midpoint of a direct line between the patella and hip joint. The value before the injection was set to 100% and relative variation of muscle thickness was expressed as mean ± standard error of the mean (SEM). To analyse significance, a paired t-test was performed. A *P* level < 0.05 was defined as significant.

## Results

### Side effects and tolerability

All dogs tolerated the injection procedure well. No negative side effects occurred during or following the injection of TeNT. None of the dogs suffered from focal or generalized muscle cramps.

### Effect of TeNT on stance and gait

Using the modified functional scoring system in dogs (mFSSD) [19] all dogs improved their weight bearing and pelvic limb gait after the injection of TeNT compared with baseline gait function before the injection. This improvement was present after a 4-week interval and still existing after a 6-week interval. However, in some of the dogs (dog #1, dog #2, dog #4a) a score decrease compared with the 4-week interval was observed. The improvement was present in most of the dogs at both sides (dog #3, dog #4a, and dog #4b) whereas dog #1 only improved on the right side. Dog #2, which was injected only at the paretic right side, showed regular pelvic limb movement at the non-paretic left side at baseline (Table 3).

**Table 3:** Effect of TeNT on gait function.

Dog no.	Injection day		Week 4		Week 6	
	Left	Right	Left	Right	Left	Right
#1	0	0	0	5	0	3
#2	14	8	14	12	14	11
#3	5	6	10	10	10	10
#4a	4	3	5	5	4	4
#4b	5	5	9	9	9	9

Using the functional scoring system in dogs (mFSSD, score 0–14) gait function before TeNT injection as well as 4 and 6 weeks after the injection was evaluated separately for both sides. In the mFSSD stands a higher score for a better gait function or rather improved recovery which means a normal pelvic limb gait is scored with 14. Dog #4 was treated twice with an interval of 16 weeks between (dog #4a and dog #4b). Dog #2 exhibited only a paresis on the right side and was solely injected on the affected side. The left side showed normal gait function (score 14).

#### Individual effect of TeNT on stance and gait of dog #1

This dog was paraplegic and proprioception as well as nociception were absent below the lesion level (L1, Table 1). The animal was not able to stand or walk leading to a gait score (mFSSD) of 0 on both sides.

**Fig. 1:**
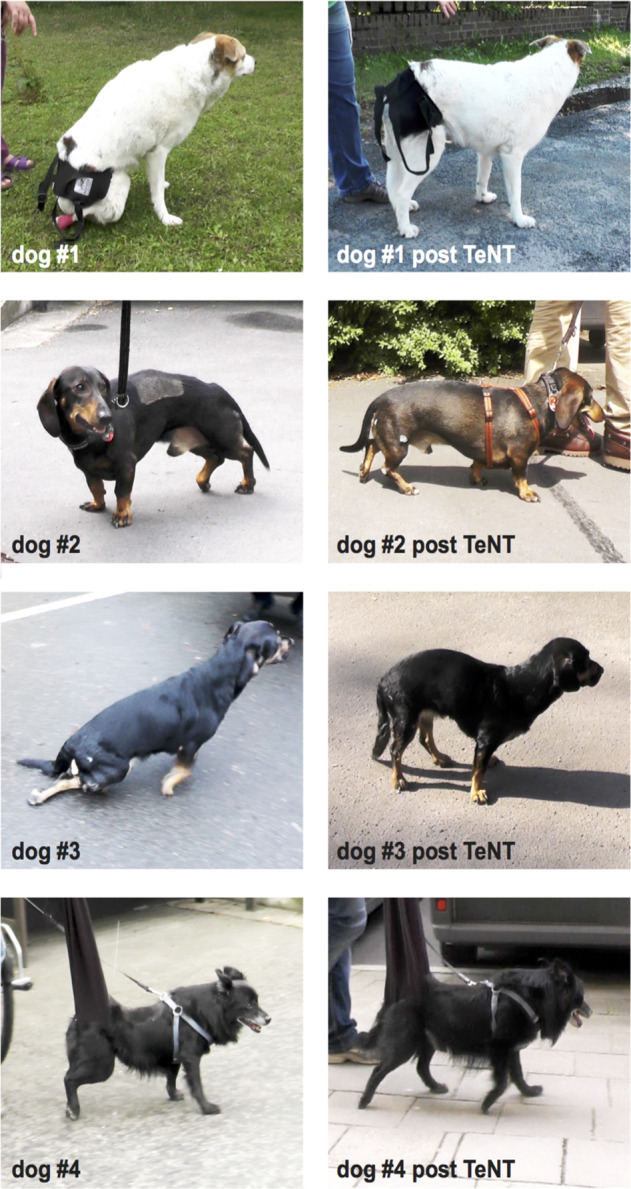
Four TeNT treated dogs before (left pictures) and after the injections (right pictures). Dog #1, a paraplegic dog (left picture), became able standing unsupported for up to 30 s 4 weeks after the injection of TeNT (right picture). Dog #2, a monoparetic dog in term of a drop paw (left picture) could walk, after 4 weeks post injection of TeNT, without rolling the paw over (right picture). Dog #3, a paraparetic dog, initially showed no weight-bearing protraction of the left pelvic hind limb but voluntary extension of the hip and knee joint (left picture). Four weeks after the injection with TeNT the dog was able to perform 10–15 weight-bearing steps, then it collapsed and walked again (right picture). Dog #4, a paraparetic dog that performed no weight-bearing protraction of the pelvic hind limb before the injection (left picture), was, after 4 weeks, able to walk with the help of a scarf bearing ~50% body weight (right picture).

After TeNT injection gait score improved after 4 weeks on the right side to 5 whereas the left side persisted unimproved (score 0). After two more weeks, the pelvic limb gait on the right side declined to a score of 3 (Table 3). At weeks 4 and 6, dog #1 was able to stand unsupported for up to 30 s (Fig. 1). However, when turning the head or stepping with the front limbs, the hind limbs collapsed. In addition, the right hind limb actively supported crawling by pushing-off in the late stance phase in the sense of an extension of the hip and knee joint. Even with full-weight support walking was not possible.

#### Individual effect of TeNT on stance and gait of dog #2

Dog #2 suffered from a distal monoparesis of the right hind limb, i.e. a drop paw. Vegetative functions and nociception below the lesion level were preserved; however, proprioception was incomplete (Table 1). Before TeNT injection, the dog was able to weight-bear with the pelvic limb muscles <50% of the time. It either rolled the right paw over or lifted it completely off the ground (Fig. 1). As a result, the skin of the dorsal surface of the right paw was chronically wounded. The initial mFSSD gait scores were 14 (left) and 8 (right).

After TeNT injection, the gait score on the treated right side improved to 12 at week 4 with a little decrease at week 6 (score 11). The left unaffected side was not injected with a correspondingly unaltered normal score [14] during the observation period (Table 3). After the injection, the animal was able to perform full weight-bearing steps in a timely correct manner more than 50% of the time. It was able to trot without rolling the paw over and it now positioned its right paw correctly during the stance phase (Fig. 1). The skin lesion of the paw healed during both follow-ups.

#### Individual effect of TeNT on stance and gait of dog #3

This animal suffered from a left-accentuated paraparesis and was incontinent for stool and urine. Nociception was absent below the lesion level (L1) whereas proprioception was incomplete (Table 1). Before the TeNT injection, dog #3 was able to stand for <3 s. The dog was able to put some weight on the right paw <10% of the time and was not able to perform a step. During crawling it was only able to extend the hip or knee actively (Fig. 1). The initial functional gait scores were 5 (left) and 6 (right).

After TeNT injection, the gait scores improved to 10 for both pelvic limbs. This effect was detectable after 4 and 6 weeks following the injection (Table 3). Accordingly, after the TeNT treatment the dog was able to stand for more than 1 min and to perform 10–15 full-weight-bearing steps consecutively at weeks 4 and 6 (Fig. 1).

#### Individual effect of TeNT on stance and gait of dog #4

Dog #4 had a right-accentuated paraparesis and was incontinent for stool and urine. Nociception was completely absent below the lesion level (Th13) whereas proprioception was incomplete (Table 1). Before the initial TeNT injection dog #4a could not stand and gait scores were 4 (left) and 3 (right). Under full-weight support, the left hind limb altered between a timely correct stance and swing phase and flaccidity. The right hind limb was constantly flaccid (Fig. 1).

Following the injections, the pelvic limb gait improved after 4 weeks to mFSSD 5 at both sides. After 2 more weeks, an improved score of bilateral mFSSD 4 was still detectable (Table 3). Walking without body weight support remained impossible, though the level of required support decreased. The left hind limb consistently performed regular gait cycles, while the more affected right hind limb only intermittently performed regular gait cycles.

Dog #4a was the only one which was injected for a second time (dog #4b). Before the second TeNT injection 16 weeks after the first TeNT injection the gait score was still slightly improved (mFSSD 5 at both sides) as compared with the baseline score (mFSSD 4 at the left side and mFSSD 3 at the right side) before the first treatment. After the second treatment, dog #4b further improved its gait score (mFSSD 9 at both sides), which was apparent after 4 as well as 6 weeks following the second TeNT injection (Table 3). This dog became able to stand for about 30 s and remained standing, even when turning its head. It started crawling and lifted its rear part to perform up to ten weight-bearing steps (Fig. 1).

### Effect of TeNT on muscle thickness

**Fig. 2:**
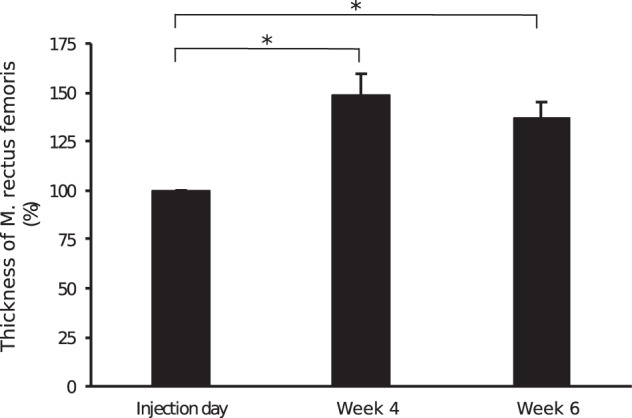
Effect of TeNT on thickness of M. rectus femoris. Relative thickness of the rectus femoris muscle at 4 and 6 weeks following the TeNT injections was quantified. The muscle thickness before the injection was set as 100%. The values of right and left M. rectus femoris after the 5 performed TeNT injection procedures (dog #1–dog #4b) were pooled (*n* = 9). The asterisks indicate significant results between the muscle thickness after 4 as well as after 6 weeks and baseline thickness before TeNT injection. Data are represented as mean ± SEM.

The individual muscle thickness of all rectus femoris muscles (*n* = 9) increased compared with individual baseline muscle thickness before injection of TeNT (baseline: 100%). This effect was maximal at the first visit 4 weeks after the injections (148.7% ± 10.9%). At the second visit, 6 weeks following TeNT injection, this increase was still present but declined compared with the 4-week interval (137.1% ± 7.9%). Paired *t*-test revealed significant results for both time intervals (*p < 0.05; Fig. 2).

Dog #2 was only injected at the right side and developed at the affected side an increase of muscle thickness by 136.2%. Thus, after the 4-week interval the thickness of the TeNT treated paretic muscle (2.41 cm) reached almost the value of the non-affected and non-injected side (2.74 cm; Table 4).

**Table 4:** Effect of TeNT on individual muscle thickness.

Dog no.	Injection day		Week 4		Week 6	
	Left	Right	Left	Right	Left	Right
#1	1.88 cm (100%)	2.02 cm (100%)	2.47 cm (131.4%)	3.68 cm (182.2%)	2.26 cm (120.2%)	3.56 cm (176.2%)
#2	2.75 cm	1.77 cm (100%)	2.74 cm	2.41 cm (136.2%)	2.75 cm	2.38 cm (134.5%)
#3	1.34 cm (100%)	1.56 cm (100%)	2.73 cm (203.7%)	2.89 cm (185.3%)	2.25 cm (167.9%)	2.47 cm (158.3%)
#4a	2.41 cm (100%)	2.32 cm (100%)	3.22 cm (133.6%)	3.08 cm (132.8%)	3.00 cm (124.5%)	2.79 cm (120.3%)
#4b	3.05 cm (100%)	2.81 cm (100%)	3.66 cm (120.0%)	3.18 cm (113.2%)	3.61 cm (118.4%)	3.20 cm (113.9%)

Individual thickness of both rectus femoris muscles of each dog (dog #1–dog #4) was measured by ultrasound at mid thigh level and is specified in cm. Thickness at the injection day was set as 100% and relative changes of thickness 4 and 6 weeks after TeNT injections were calculated. Dog #4 was treated twice (dog #4a and dog #4b) with an interval of 16 weeks in between. Since dog #2 presented with a monoparesis on the right side, accordingly the right pelvic limb was treated and only relative changes in muscle thickness at this side were calculated. The ‘Left’ column shows the diameter of the left side.

## Discussion

In this case series, we describe the first use of intramuscular TeNT for the treatment of motor symptoms of SCI in dogs. In all four dogs, which received low-dose TeNT injections into the paretic muscles of the hind limbs, either stance or gait improved for several weeks. In addition, TeNT treatment restored atrophy of the affected hind limb muscles.

All four dogs had received a spinal decompression surgery and subsequent physiotherapy including locomotor training before TeNT treatment without beneficial effects on their motor symptoms. Following the TeNT injections, the paraplegic dog (dog #1) was able to stand unsupported for up to 30 s and started crawling actively with its right pelvic limb. Both paraparetic dogs became ambulatory without support after the first injection (dog #3) or after the second injection (dog #4b), respectively. Also, the monoparetic dog (dog #2) experienced a recovery from its drop paw and walked in a more physiological manner after low-dose TeNT injections.

To evaluate the effects of stance and gate we made use of the well-established FSSD [19]. Since the dogs’ hind limbs were differently affected we modified the FSSD so that each hind limb was rated separately (mFSSD). Nonetheless, we did not made use of the scoring system (0–10) of Lee et al. [20] that developed also a modified functional scoring system in dogs since the scale (0–14) of Olby et al. [19] corresponds better to the clinical presentation of the dogs. The scoring system of Lee et al. [20] was designed to evaluate ambulatory dogs that can perform at least four steps. Some of our dogs (dogs #1, #3, and #4) were non-ambulatory prior to the injections so that their scores would have been below 6 and the scoring system of Olby et al. [19] provided a higher resolution in grading the specific impairments of the dogs.

Our results must, however, be interpreted cautiously. The intervals between acute disc herniation and surgery varied between the dogs from 1 week to 1 year. The interval between surgery and the TeNT treatment as well ranged from 1 week to almost 3 years. Dog #2, which had a short time interval between SCI and TeNT injection, could have improved spontaneously or as a result from the surgery. Moreover, in dog #4 with a 7-week interval between SCI and TeNT injection, partial spontaneous recovery cannot be ruled out. However, those dogs with a greater time between their SCI and TeNT injections (dog #1: 83 weeks; dog #3: 157 weeks) were not expected to recover spontaneously [17, 19]. Effects on motor symptoms are therefore most likely due to the TeNT treatment.

TeNT inhibits the fusion of vesicles containing the inhibitory neurotransmitters glycine and GABA and thereby leads to disinhibition at spinal level [9]. It is assumed that this unique mode of action is responsible for the substantial increase in activity and tone within the injected hind limb muscles of our dogs. However, as the dogs not only regained muscle strength, but also had restored gait cycles, further neuronal networks of the spinal cord are very likely involved. In addition to the mere facilitation of α-motor neurons, TeNT may also have disinhibited the central pattern generators (CPGs) of the mature dogs. The anatomical substrate of the CPGs is a complex network of excitatory and inhibitory interneurons within the lumbar spinal cord that coordinate cyclic hind limb movements. Physiologically, these spinal CPGs are controlled by the brainstem [21–24]. If available, sensory signals via movement of the limbs may lead to further adaptation of the movements [4, 25]. However, in animals these CPGs can be activated without any other input, in contrast to humans where some level of supraspinal control needs to be preserved [23]. Generally, CPGs can be activated by electrical stimulation [25], by pharmacological treatments [26], or by the combination of both [2, 4].

The beneficial effects of TeNT in the reported dogs with SCI highlight a possible therapeutic strategy of using TeNT to focally disinhibit spinal motor circuits within the pathophysiological concept of an unbalanced equilibrium between spinal inhibition and excitation that results from an impaired upper motor activity.

Moreover, the assumption of a re-activation of spinal CPGs via a blockade of spinal inhibitory interneurons is supported by the fact that those dogs that had incomplete proprioception before the TeNT injection (dog #2, #3, and #4) were able to perform few steps after the treatment. In contrast, the dog without any proprioception (dog #1) was only able to stand following the TeNT injection but not able to perform any steps. However, this mechanism could not easy be translated to humans since human CPGs could not only be initiated by proprioception but would also require supraspinal input [27].

In addition to the effect on stance and gait, a further prominent effect of TeNT injection was an obvious increase in mass of the injected muscles in all four dogs. This effect was confirmed by repeated ultrasound measurements of the rectus femoris muscle. Pathophysiologically, motor diseases of central origin lead to atrophy of the affected musculature [28]. Only functional electric stimulation is able to partially reverse muscle atrophy [29]. Remarkably, in dog #2 which initially presented a monoparesis, muscle size was restored almost to muscle size of the unaffected side; however, due to the short time between the SCI and the surgery it is not possible to determine the reason for this recovery.

This profound TeNT effect on muscle trophism is most likely a direct result of the increased α-motor neuron activity. Higher levels of spontaneous or reflective motor neuron activation induce an increase of skeletal muscle mass. Pharmacologic focal reversal of muscle atrophy in motor disease of central origin has not previously been described. In addition to the increase of muscle tone, this mode of action may evolve as one of the most promising TeNT effects in future clinical studies.

Safety is an important issue when TeNT effects are going to be transferred into the clinical setting. Symptoms from wound infections with *Clostridum tetani* and subsequent generalized TeNT intoxications, i.e. uncontrolled and pronounced muscle spasms or a spreading of TeNT effects to higher or lower levels than the injected [10, 11] must be sought. However, from animal studies it is known that these effects are strongly related to the applied dose [30, 31]. At higher doses, retrogradely transported TeNT spreads at the spinal level and affects motor neurons of adjacent or contralateral spinal segments. In addition, at higher doses TeNT can spread via blood circulation to distant muscles, as known from wound infections [11].

At very low doses, intramuscular TeNT results in a temporary increase in muscle tone without any spread or generalization and also without inducing painful muscle cramps [12]. In our case series, the effects of low-dose TeNT were restricted to a localized increase in muscle tone without any signs of painful muscle spasm or an unwanted spreading beyond the injected hind limbs. These results underline that intramuscular injections of low doses of TeNT are safe in dogs and may be considered as an option for therapeutic use in humans as well. Obviously, the chosen doses of the biologically validated TeNT were within a therapeutic range, for which limits still have to be determined.

Since the actual case series shows only pilot data, future animal studies are needed to further investigate clinical TeNT effects with respect to duration, effect size, and safety margin. As the induced effects could vary between different species, placebo controlled clinical studies should be performed in dogs and would certainly need to be performed in humans. Due to the high similarity of the protein structures of tetanus and botulinum neurotoxins, it would be of special interest to apply repeated TeNT injections, since botulinum neurotoxin is clinically injected quarterly to maintain the induced effects.

A further critical issue of any transfer of low-dose TeNT into clinical application is related to the fact that most people in developed countries underwent active TeNT immunization. Theoretically, specific antibodies against TeNT may prevent the effects of therapeutic intramuscular TeNT injections. However, earlier mice studies confirmed that TeNT effects were not affected by prior immunization in mice [13]. It is possible that the exceptional high affinity to the neuronal membrane of TeNT and a resulting rapid binding to motor neurons [32–34] results in a lower probability for a binding to circulating TeNT antibodies. It has furthermore been speculated that antibodies may neutralize only that minor proportion of TeNT, that get into the bloodstream, thus preventing generalized symptoms [13]. Further studies should address this issue which is specific for human application.

## Data Availability

All data generated or analysed during this study are included in this published article.
